# Directed self-assembly of a two-state block copolymer system

**DOI:** 10.1186/s40580-018-0156-z

**Published:** 2018-09-27

**Authors:** Hyung Wan Do, Hong Kyoon Choi, Karim R. Gadelrab, Jae-Byum Chang, Alfredo Alexander-Katz, Caroline A. Ross, Karl K. Berggren

**Affiliations:** 1 Department of Electrical Engineering and Computer Science, Massachusetts Institute of Technology, Cambridge, MA 02139 USA; 2 Department of Chemical and Biomolecular Engineering, Korea Advanced Institute of Science and Technology, Daejeon, South Korea; 3 Department of Materials Science and Engineering, Massachusetts Institute of Technology, Cambridge, MA 02139 USA; 4 Division of Advanced Materials Engineering, Kongju National University, Cheonan, South Korea; 5 Department of Biomedical Engineering, Sungkyunkwan University, Seoul, South Korea

**Keywords:** Block copolymers, Self-assembly, Graphoepitaxy, Nanostructures, Lithographic confinement

## Abstract

In this work, ladder-shaped block copolymer structures consisting of parallel bars, bends, and T-junctions are formed inside square confinement. We define binary states by the two degenerate alignment orientations, and study properties of the two-state system. We control the binary states by creating openings around the confinement, changing the confinement geometry, or placing lithographic guiding patterns inside the confinement. Self-consistent field theory simulations show templating effect from the wall openings and reproduce the experimental results. We demonstrate scaling of a single binary state into a larger binary state array with individual binary state control.

**Electronic supplementary material:**

The online version of this article (10.1186/s40580-018-0156-z) contains supplementary material, which is available to authorized users.

## Introduction

Block copolymer self-assembly in thin films can spontaneously generate periodic nanoscale patterns such as hexagonal arrays of dots or parallel lines, which have been proposed for applications such as nanoporous filtration membranes [1, 2], plasmonic structures [3, 4], integrated circuit fabrication [5–7], and magnetic storage media [8–10]. Many of these applications require the nanoscale features to have long-range order or to form specific non-periodic structures with low defect density. Directed self-assembly (DSA) addresses these issues by using graphoepitaxial [11–14] and/or chemoepitaxial [15–19] templates, fabricated by conventional lithography techniques, to guide the self-assembly of thin films of block copolymers. Various microelectronic device-oriented features such as concentric rings, bends, jogs, terminations, and T-junctions have been made, and these patterns have subsequently been transferred into functional materials to fabricate structures such as metal nanowire ring arrays [20–22] or parallel fins for field-effect transistors [7, 23, 24].

Common templates used for DSA include one-dimensional features (trenches or chemical stripe patterns), or two-dimensional features (pits or chemically patterned regions). Although the templating effect from trench confinement has been well studied [25–27], two-dimensional templates provide a wider set of geometries to guide block copolymer self-assembly, and can lead to formation of multiple degenerate structures. For example, concentric ring structures have been self-assembled inside symmetric confinements [20, 21], and we recently demonstrated nanoscale Archimedean spirals with specific chirality formed inside circular pits [28]. By studying such block copolymer systems that have energetic degeneracy, graphoepitaxial pattern control inside two-dimensional templates can be better understood for lithography applications. Moreover, by assigning different states or bits to the two degenerate morphologies, the resulting block copolymer patterns could act as a physical read-only memory.

This article describes DSA of block copolymer films within templates of different polygonal shapes. In square templates, two degenerate morphologies can form, and the presence of junctions between the templates breaks the degeneracy. We describe the properties of the binary states including distribution, correlation, and defect tolerance. We present three methods for controlling the binary state orientations and demonstrate the propagation of a single binary state into a larger array with orientation control.

## Results and discussion

We first demonstrate the morphologies of a block copolymer film within polygonal templates. The block copolymer is a 45.5 kg/mol cylindrical morphology poly(styrene-*block*-dimethylsiloxane) (PS-*b*-PDMS) (SD45). Thin films of SD45 microphase separate into a layer of PDMS cylinders with in-plane orientation surrounded by a PS matrix, and a wetting layer of PDMS at the air interface. Electron-beam lithography was performed using a hydrogen silsesquioxane (HSQ) resist on silicon substrates to fabricate topographic features of various geometries. The topographic templates were chemically functionalized with a hydroxyl-terminated PS brush. SD45 block copolymer was spin coated onto the substrate to a thickness of 27 nm, solvent annealed in a vapor of toluene and heptane, and reactive-ion etched to reveal a pattern consisting of oxidized PDMS cylinders.

**Fig. 1:**
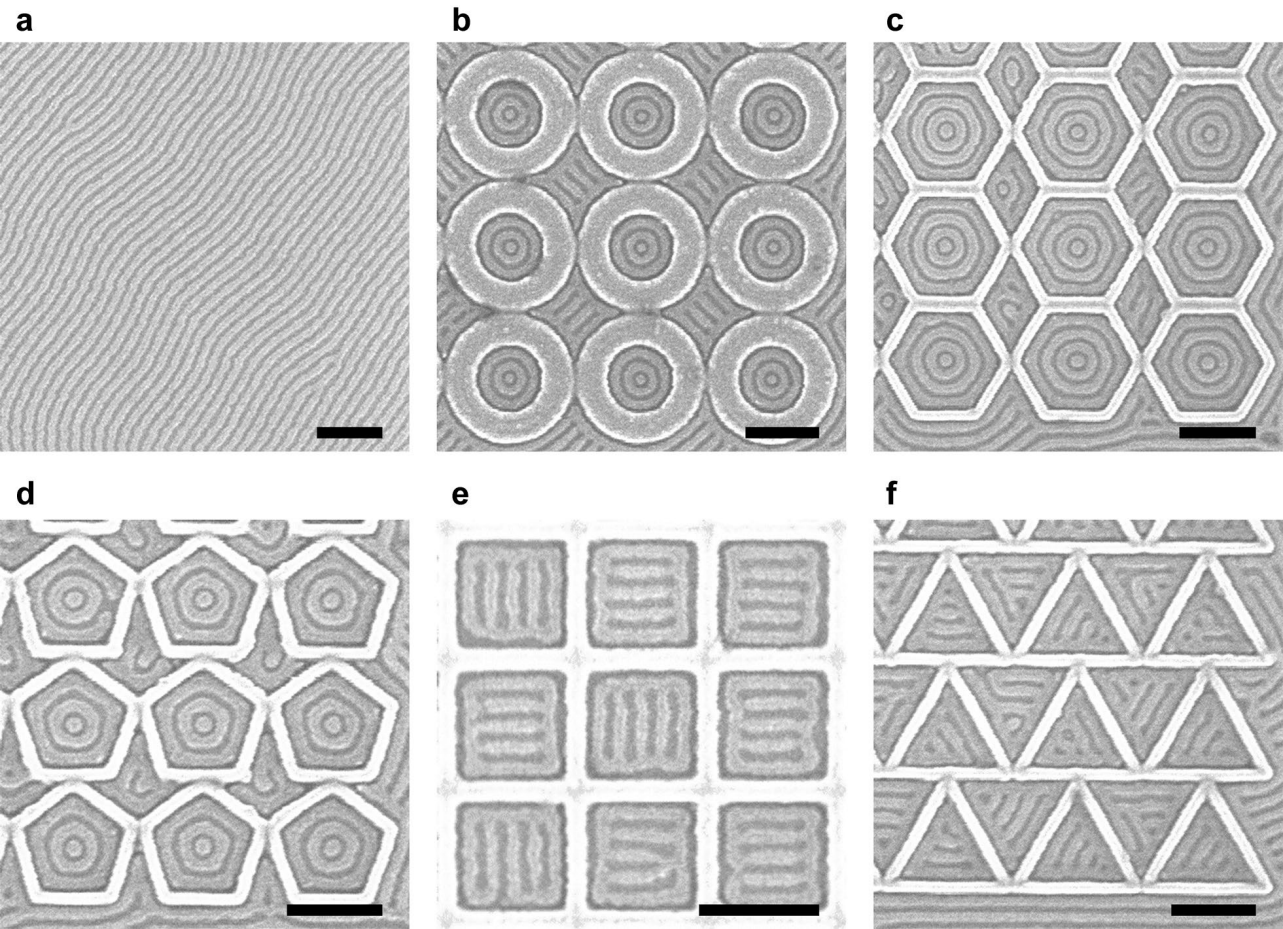
Scanning electron microscope (SEM) images of untemplated and templated block copolymer patterns. The HSQ templates were functionalized with the majority PS block. **a** Untemplated PDMS cylinders with *L*_0_ = ~ 36 nm. **b** One-state system with concentric rings inside circular confinement. Radius was 2.4*L*_0_. **c** One-state system inside hexagonal confinement. Apothem was 3.5*L*_0_. **d** One-state system inside pentagonal confinement. Apothem was 2.5*L*_0_. **e** Two-state system with degenerate ladder-shaped structures inside square confinement. Apothem was 2.4*L*_0_. **f** Three-state system inside triangular confinement. Apothem was 1.8*L*_0_. The radius and apothem were measured by subtracting brush thickness from confinement dimensions. Scale bars, 200 nm

Figure 1 shows an example of oxidized PDMS patterns without any template (Fig. 1a) and within polygonal confinement (Fig. 1b–f). On an untemplated substrate, the periodicity of the PDMS cylinders (*L*_0_) was ~ 36 nm. As shown in Fig. 1b–d, the PDMS cylinders formed a one-state system of concentric rings inside circular, hexagonal, and pentagonal confinement. However, the PDMS cylinders formed a two-state system inside square confinement and a three-state system inside triangular confinement (Fig. 1e, f). For both confinements, bars parallel to one of the sides were formed inside an outer ring, creating 90° T-junctions for square confinement and 60° Y-junctions for triangular confinement. As the interior angle is decreased, high deformation is imposed on the microdomains at the corners [15, 17], which is relieved by transitioning to a pattern of parallel bars instead of concentric rings. For square confinement, orientation of the parallel bars was restricted to either the horizontal or vertical direction, and these degenerate states are defined as the basis states of the system.

**Fig. 2:**
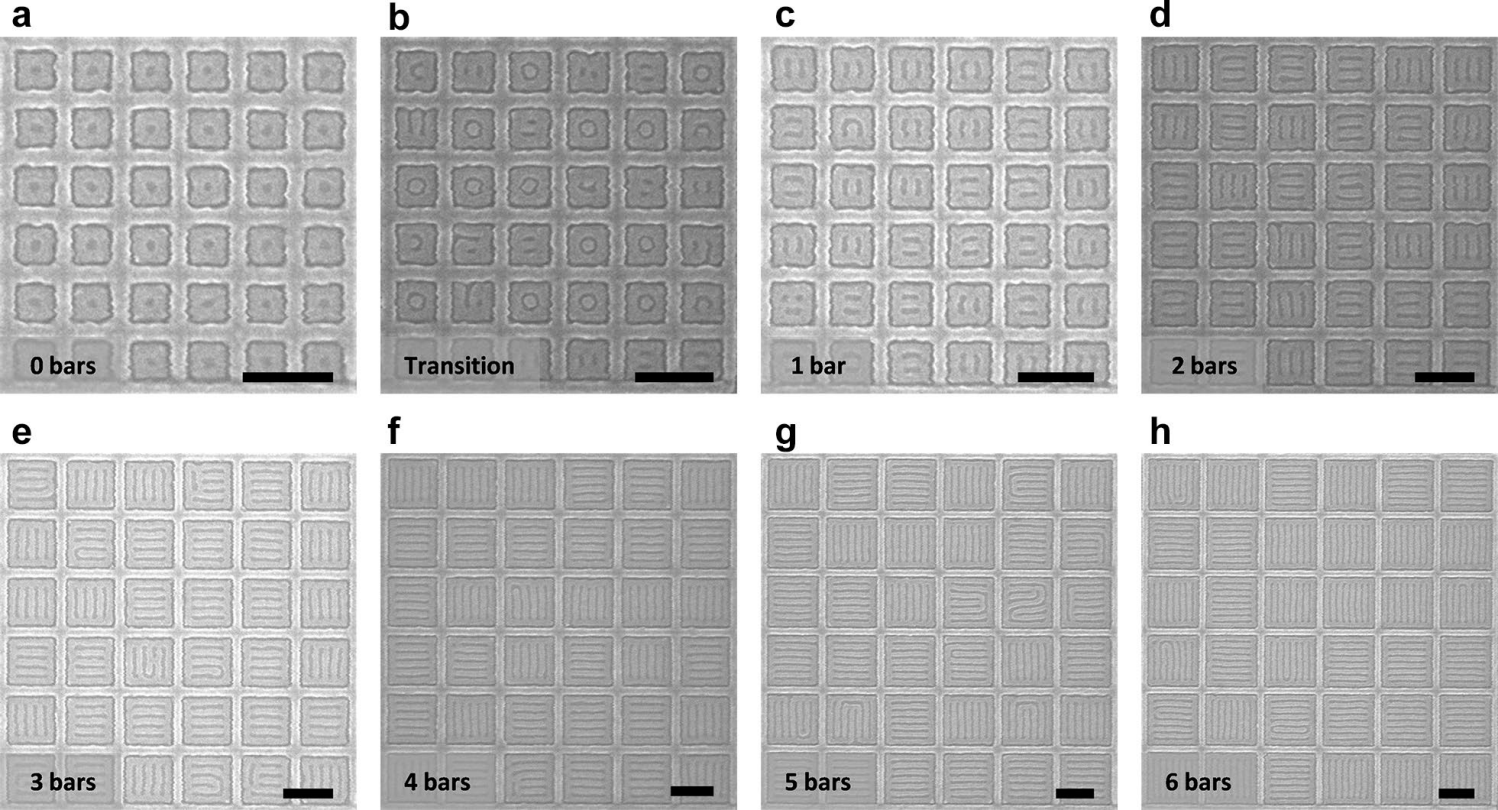
SEM images of ladder-shaped block copolymer patterns inside square confinement. **a**, **c**–**h** Show commensurate conditions while **b** shows transition between a one-state system (**a**) and a two-state system (**c**) inside incommensurate confinement dimensions. Width of the square confinement was **a** 2.2*L*_0_, **b** 2.8*L*_0_, **c** 3.0*L*_0_, **d** 4.1*L*_0_, **e** 5.1*L*_0_, **f** 6.1*L*_0_, **g** 7.1*L*_0_, and **h** 8.1*L*_0_ (*L*_0_ = 36 nm). Depending on the confinement width, 0–6 parallel bars were formed inside an outer ring. Scale bars, 200 nm

We focus our study on the square confinement since it resulted in a well-defined two-state system with 90° bends and T-junctions. Commensurability is achieved when the width of the confinement minus the brush thickness is equivalent to an integer multiple of *L*_0_. Figure 2 shows the ladder-shaped block copolymer patterns formed inside square templates, with the number of parallel bars increasing with confinement dimensions. The smallest templates produced a one-state system consisting of a single ring (Fig. 2a), then a PDMS sphere was formed inside the outer ring as the confinement dimension was increased between 2*L*_0_ and 3*L*_0_ (Fig. 2b). In this regime, both ladder-shaped structures (two-state system) and concentric ring structures (one-state system) were observed. With increasing dimensions the interior spheres were either horizontally or vertically connected to the outer ring resulting in a two-state system, then an additional bar was formed inside the outer ring (Fig. 2c–h). For larger incommensurate templates, ladder-shaped structures were still produced, but the number of parallel bars varied by one from structure to structure.

To show that the two basis states are degenerate, we created arrays of 10 by 10 square templates and measured the distribution of horizontally aligned and vertically aligned ladder-shaped structures. For simplicity, we defined horizontal alignment as the ‘0’ state and vertical alignment as the ‘1’ state. Among 600 examined structures, 51.5% were in 0 state and 48.5% were in 1 state, forming with essentially equal probability. For a null hypothesis *H*_0_: *p* = 0.5 and alternative hypothesis *H*_1_: *p* ≠ 0.5 where *p* denotes ratio of 1 state, the Z-test statistic was 0.735. The corresponding *p*-value was 0.462, and we failed to reject the null hypothesis at 5% level of significance. In addition, the binary states had tolerance to defects in the sense that even with defects present the patterns could be assigned as 0 or 1 (Additional file 1: Figure S1a).

Next, we investigated whether the binary state of the four neighbors was correlated with the binary state of the surrounded square. For each square not positioned on the boundary of the square array, there were four adjacent squares as indicated by the red dashed line (Additional file 1: Figure S1a). The normalized mean state–state correlation is

$$\rho = \frac{{\mathop \sum \nolimits_{i\sim j} s_{i} s_{j} }}{{\mathop \sum \nolimits_{i\sim j} |s_{i} s_{j} |}} = 0.00078$$

where $s_{k} = + 1$ for 1 state, $s_{k} = - 1$ for 0 state, and the sum was taken over every pair of adjacent states. The negligible value of correlation suggests that there is no nearest-neighbor influence.

To investigate the correlation between isolated pairs of adjacent square confinements in samples shown in Additional file 1: Figure S1b, we define *n*_*XY*_ as the number of cases where the left binary state is *X* (0 or 1) and the right binary state is *Y* (0 or 1). For 576 structures, the resulting counts were *n*_00_ = 144 (25.0%), *n*_01_ = 137 (23.8%), *n*_10_ = 143 (24.8%), and *n*_11_ = 149 (25.9%) with 3 (0.5%) defects. The *ϕ* coefficient calculated as

$$\phi = \frac{{n_{00} n_{11} - n_{01} n_{10} }}{{\sqrt {\left( {n_{00} + n_{01} } \right)\left( {n_{00} + n_{10} } \right)\left( {n_{01} + n_{11} } \right)\left( {n_{10} + n_{11} } \right)} }} = 0.023$$

was close to zero, indicating negligible association between two adjacent states in isolated pairs of square confinements. A similar set of samples made with a template wall height of 30 nm instead of 42 nm yielded *ϕ* = − 0.03, again indicating negligible association. For wall height below 30 nm, the PDMS cylinders crossed the walls leading to poorly defined block copolymer structures within the templates.

**Fig. 3:**
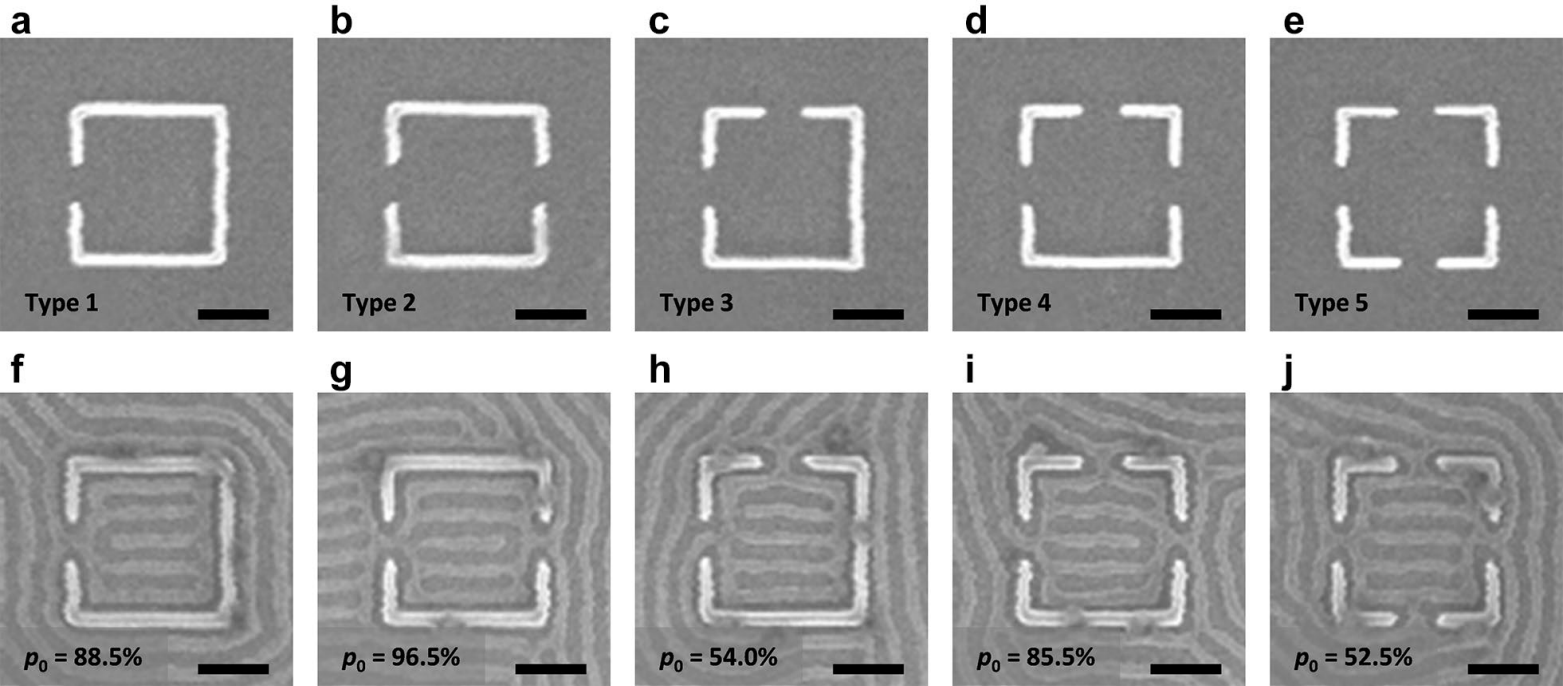
SEM images of square confinement with openings and the resulting block copolymer patterns. Ratio of 0 state (denoted by *p*_0_) for each type was measured in a large array. Binary states were determined based on the number of horizontal and vertical openings. **a**, **f** Square confinement with one horizontal opening. Preferential horizontal alignment (0 state) was observed. **b**, **g** Square confinement with two horizontal openings on non-adjacent sides. Stronger preferential horizontal alignment was observed. **c**, **h** Square confinement with two openings on adjacent sides. Alignment in both directions was observed with equal probability. **d**, **i** Square confinement with three openings. Preferential horizontal alignment was observed. **e**, **j** Square confinement with four openings. Alignment in both directions was observed with equal probability. These results demonstrate the templating effect from openings. Scale bars, 200 nm

Having established the non-interacting binary-state system described above, we now discuss methods to control the alignment of the states. A simple method for controlling the orientation of the binary states is by creating openings in the confinement. Figure 3 shows five possible types of 5*L*_0_ wide square confinements with one to four 1*L*_0_ wide openings on the sides of the confinement. The fraction of 0 (horizontal cylinders) states was measured for each type of confinement. When there were equal numbers of openings on the top and bottom sides and left and right sides as in type 3 and 5 configurations (Fig. 3c, e), there were equal numbers of 1 and 0 states. However, when there were more openings on the left/right sides than top/bottom sides as in type 1, 2, and 4 configurations (Fig. 3a, b and d), the 0 state was favored. The highest yield of a preferential alignment was achieved in the type 2 configuration where both openings were in the same direction. When there was one less opening in the same direction (type 1) or one more opening in the other direction (type 4), the yield was decreased by ~ 10%. As the number of openings around the square template was increased, there was less templating effect from the confinement and more defects were formed, but the binary state was still evident (Additional file 1: Figure S2). For arrays of square confinements with four openings, the normalized mean state–state correlation was *ρ* = − 0.00207, suggesting no nearest-neighbor influence even with openings present between states.

**Fig. 4:**
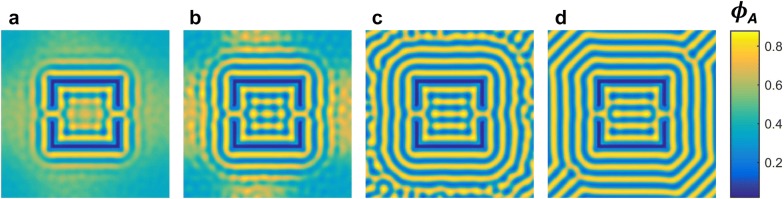
SCFT simulations showing the evolution of the polymer self-assembled pattern. **a** Early stages of the simulation show a wetting layer is formed of the inner and outer surface of the walls while the polymer is still disordered in the region surrounding the template. A junction is formed at every opening in the template wall, connecting the inner and outer wetting layers. **b** An ordering front is propagating away from the walls creating a series of concentric squares. Polymer microdomains are nucleated adjacent to the bends in the inner wetting layer caused by the opening in the walls. **c** Bridging of the polymer domains creates a horizontal stripe between the two openings. **d** Final polymer self-assembled pattern reaches 0 state as shown experimentally

Self-consistent field theory (SCFT) was used to model the effect of a wall opening as a templating method on the final state of the system. We focused on the type 2 template that had the highest preference for one binary state. Although SCFT does not calculate dynamics directly, after several steps order emerges and the model is expected to resemble the evolution of the physical system. Figure 4a–d show the evolution of the SCFT model starting from a random state. The high attraction at the walls initiated a uniform wetting layer (Fig. 4a) from which an ordering front propagated away from the walls resulting in concentric squares surrounding the template. Two symmetric junctions were formed at the openings in the template walls (Fig. 4b) connecting the inner and outer wetting layers of the template; however, the 1*L*_0_ wide openings became blocked isolating the inner polymer domains. In addition, the small outward curve of the wetting layer at the openings caused two horizontally aligned polymer domains to form which eventually bridge to create a horizontally disconnected polymer stripe, in remarkable agreement with experiment (Fig. 4c). The presence of this stripe biases the system into the 0 state (Fig. 4d). Additional file 1: Figure S3 shows that the templating effect of the junctions was observed for various simulation conditions (*χN* and strength of wall preferentiality) where the alignment of polymer domains was consistently parallel to the wall openings. On the other hand, the junction connecting the inner and outer wetting layers was only stable for low *χN* and strong wall attraction. This suggests the robustness of this templating approach to direct the system into a particular state. Based on the strong templating effect from the openings, we expect to observe preferential alignment even when the opening position is changed, as long as the Y-junction is properly formed.

**Fig. 5:**
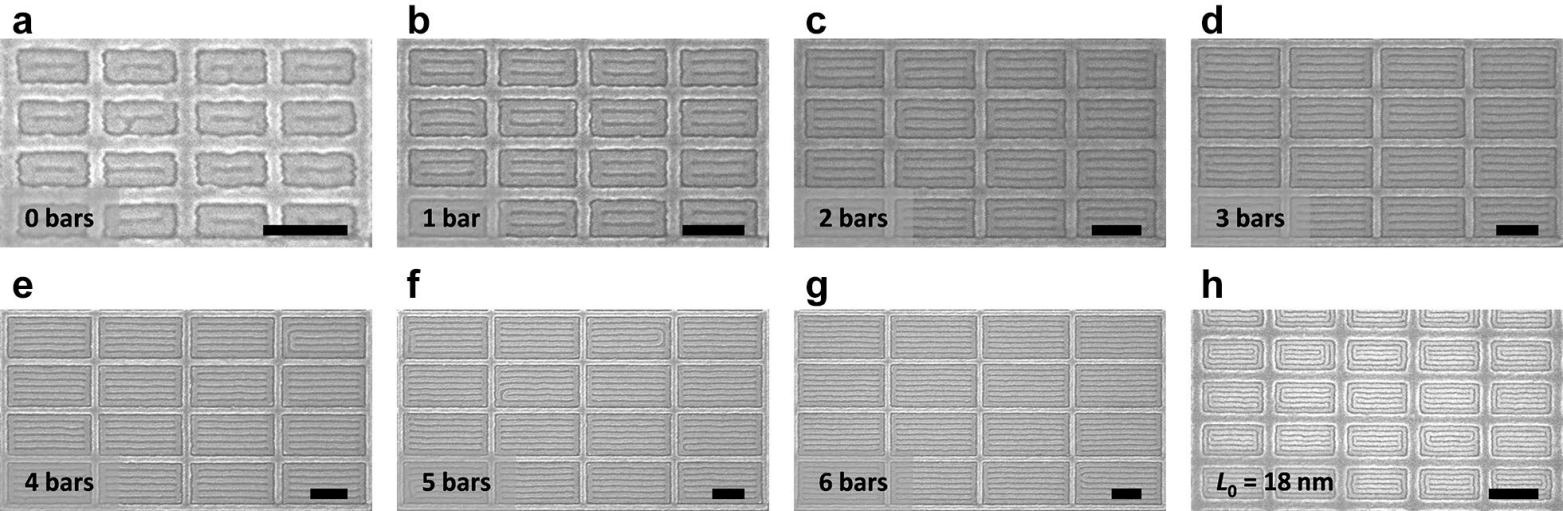
SEM images of aligned ladder-shaped block copolymer patterns inside rectangular confinement. Aspect ratio was 2:1. Vertical width of the rectangular confinement was **a** 2.0*L*_0_, **b** 3.1*L*_0_, **c** 4.1*L*_0_, **d** 5.1*L*_0_, **e** 6.1*L*_0_, **f** 7.0*L*_0_, and **g** 7.9*L*_0_ for SD45 (*L*_0_ = 36 nm). For **h**, 16 kg/mol PS-*b*-PDMS with *L*_0_ = 18 nm was used. Parallel bars were formed in the horizontal direction (0 state) to minimize the number of T-junctions. Scale bars, 200 nm

Next, we show that the orientation of the binary states can be also controlled by changing the confinement to a rectangular shape. Figure 5 shows ladder-shaped block copolymer patterns formed inside rectangular confinements with an aspect ratio of 2:1. Similar to the square confinement, we were able to accurately control the number of parallel bars using confinement dimensions. When the vertical confinement width was commensurate with *L*_0_, the horizontal confinement width was also commensurate with *L*_0_ since the aspect ratio was an integer. However, we observed only 0 state at these conditions.

When the horizontal and vertical confinement widths are equal to 2*nL*_0_ and *nL*_0_, respectively, a ladder-shaped structure in the 0 state results in 2*n* − 4 T-junctions (*n* is an integer). On the other hand, a ladder-shaped structure in the 1 state results in 4*n* − 4 T-junctions. Since T-junctions are energetically unfavorable [29–31], the 0 state was favored over the 1 state to minimize the number of T-junctions. The preferential alignment can also be understood as the longer sidewall having a stronger templating effect compared to the shorter sidewall, analogous to the perpendicular orientation of lamellar morphology PS-*b*-PDMS observed within deep trenches functionalized with a preferential sidewall brush and a neutral bottom surface [32].

In Additional file 1: Figure S4a, the horizontal and vertical dimensions of the confinement were approximately commensurate, 4.1*L*_0_ and 2.9*L*_0_, respectively, and the structure formed two T-junctions instead of four T-junctions. Non-integer aspect ratios in which only the shorter dimension satisfies the commensurability condition can also be used to further promote alignment (Additional file 1: Figure S4b). As the aspect ratio increases, the confinement approximates a trench leading to well-ordered microdomains parallel to the sidewalls.

This approach was extended to fabricate aligned T-junctions with sub-10-nm spacing using a 16 kg/mol cylindrical morphology PS-*b*-PDMS with *L*_0_ = 18 nm, thermally annealed on a functionalized patterned substrate. As shown in Fig. 5h, sub-10-nm half-pitch ladder-shaped structures were formed inside rectangular confinement with the microdomains primarily parallel to the longer side. Unlike SD45, the ladder-shaped structures were influenced by the curvature of the corners of the template due to the smaller *L*_0_, and a complete ring was formed between the confinement and the ladder-shaped structure.

Preferential alignment is observed in non-rectangular geometries such as trapezoids and isosceles triangles, as shown in Additional file 1: Figure S5. For trapezoidal confinement, T-junctions formed with desired bending angles because the microdomains aligned parallel to the longer side. Isosceles triangles typically showed preferential alignment parallel to either of the two longer sides. This produced two T-junctions whereas alignment parallel to the shorter side resulted in four T-junctions. Thus the confinement geometry determines the number of states, i.e. a three-state system in equilateral triangles (Fig. 1f), a two-state system in acute isosceles triangles or possibly a one-state system in obtuse isosceles triangles.

The third method for controlling the orientation of the binary states is by placing lithographically defined guiding patterns inside the confinement. The effect of posts, dashes, or walls has been previously studied in detail [13, 14, 33]. Additional file 1: Figure S6 shows square confinements, each with two horizontal HSQ walls where the walls were positioned a distance *L*_0_ away from the edges. Because two PDMS bars in the ladder-shaped block copolymer structures were replaced with the horizontal HSQ walls functionalized with the majority PS block, all block copolymer patterns were also horizontally aligned and set to the 0 state.

**Fig. 6:**
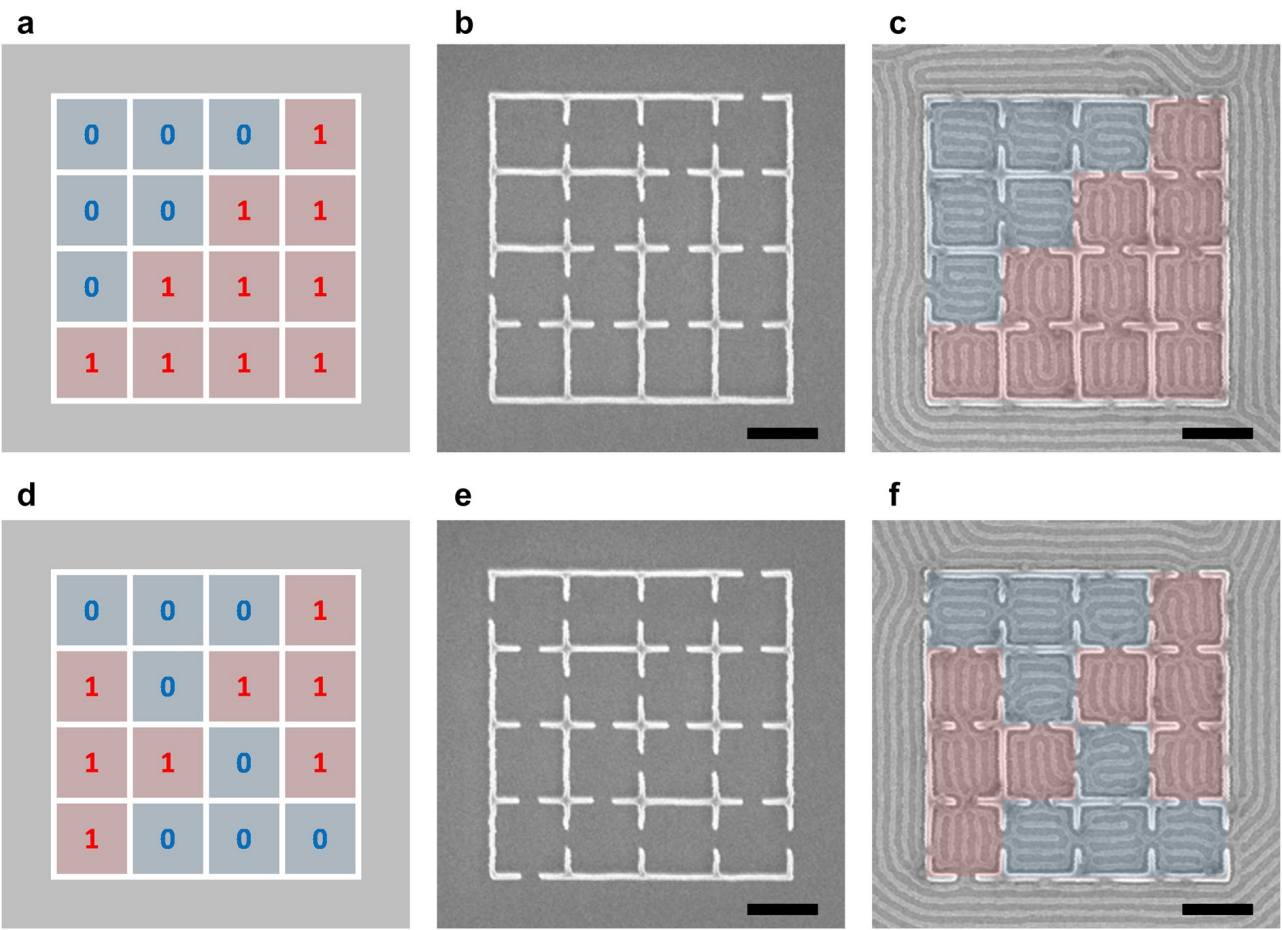
Fabrication of binary state arrays. **a**, **d** Diagram of desired 4 × 4 binary state arrays. **b**, **e** Templates fabricated by electron-beam lithography. **c**, **f** Resulting block copolymer patterns matching the desired binary states. Red indicates 1 state and blue indicates 0 state. Scale bars, 200 nm

Using the three approaches for controlling the binary state orientation, we show how the orientation can be propagated within a larger template array. We showed above that neighboring binary states are uncorrelated with each other in square confinements separated by walls. However, by creating openings in the walls, specific orientations can be programmed by selecting cells from the five types of square confinement. Figure 6 demonstrates two examples of 4 × 4 binary patterns each composed of 16 independently controlled binary states. The target patterns are shown in Fig. 6a, d, the templates in Fig. 6b, e, and the successfully produced self-assembled pattern in Fig. 6c, f. For a given target pattern of binary states, in general more than one template can be chosen. To improve yield for larger binary patterns, the second and third templating method can be used to design the template.

Additional file 1: Figure S7 shows examples of different target patterns and corresponding template designs determined by trial and error. The target patterns shown in Additional file 1: Figure S7a, c are the same as the target patterns shown in Fig. 6a, d. New templates shown in Additional file 1: Figure S7b, d were designed by changing the location of certain openings and verifying the result. However, not all target patterns are obtainable by creating openings in the walls, since the opening locations determined by the four neighbors may conflict with the opening locations required for the surrounded square. For example, a target pattern consisting of alternating states (Additional file 1: Figure S7k) cannot be obtained from creating openings. By placing guiding patterns inside the confinement where such conflict may occur, we are able to design a template that will produce the target pattern (Additional file 1: Figure S7l). In general, a template for arbitrary binary state pattern can be designed by combining the three templating approaches.

## Conclusions

The self-assembly of block copolymers inside discrete and interacting polygonal templates is investigated. Square and triangular confinement with dimensions of a few *L*_0_ produced ladder-shaped structures instead of the concentric rings seen in smaller confinements or in circular pits. In square confinement, the two degenerate orientations of the ladder-shaped structures could be considered as independently controlled binary states with tolerance to defects. The binary states were selected by either creating openings around the confinement, changing the confinement aspect ratio, or placing additional lithographic features inside the confinement. The resulting line segments, bends, and T-junctions composing the ladder-shaped structures may be useful as circuit-relevant geometries or binary information storage. Moreover, complex large-area structures can be formed by self-assembly from a sparse template with specific openings in the walls that can be written by electron-beam lithography. Although the multi-state composite structures used in our work are less than 1 μm in dimension, we expect larger sizes to yield similar result. If the binary states could be read out optically or electrically in a very large array, the block copolymer patterns could be used to physically store information in addition to functioning as a lithography mask. Future work will involve designing of a two-state system with nearest-neighbor interactions. If an output state can be controlled by lithographically defined input states, the two-state system might even be able to perform self-assembly based computation.

## Methods

### Template fabrication

The topographic templates were fabricated using electron-beam lithography with HSQ resist. A silicon substrate was spin coated with 42-nm-thick HSQ film (XR-1541 2% solids, Dow Corning). The thickness was determined by ellipsometry. A Raith 150 electron-beam lithography system operated at 30 kV acceleration voltage was used to expose topographic features with various geometries. After exposure, the samples were developed in a 24 °C high contrast salty developer (1% NaOH and 4% NaCl in de-ionized water) for 4 min, rinsed in de-ionized water for 3 min, and blow dried with N_2_ gas [34]. Template dimensions were inspected by SEM imaging. Templates for 16 kg/mol PS-*b*-PDMS were fabricated using 30-nm-thick HSQ film.

### Block copolymer self-assembly

To make the templates attractive to the majority PS block, the templates were chemically functionalized with a hydroxyl-terminated PS brush (11 kg/mol, Polymer Source Inc.) by spin coating 1% brush solution in propylene glycol monomethyl ether acetate (PGMEA) and annealing the samples in a vacuum oven at 170 °C for 14 h. The samples were rinsed with toluene for 1 min after annealing to remove excess PS brush. The resulting thickness of the PS brush bonded to the substrate was 5 nm. Next, 2% PS-*b*-PDMS (*M*_w_ = 45.5 kg/mol, *f*_PDMS_ = 32%, PDI = 1.08, Polymer Source Inc.) solution in PGMEA was spin coated onto the templated substrate. The resulting film thickness was 27 nm. The samples were solvent annealed using a 5:1 mixture of toluene and heptane at room temperature for 5 h. We placed the samples on a glass slide stack (0.8 cm in height) inside a crystallization dish (1.5 cm in height, 5 cm in diameter) and added 1.5 ml of the 5:1 toluene and heptane mixture. The chamber was covered with a petri dish (10 cm in diameter). During the 5 h annealing, leakage of solvent vapor occurred at a rate of 585 µg/min. 16 kg/mol PS-*b*-PDMS (*f*_PDMS_ = 31%, PDI = 1.08, Polymer Source Inc.) was spin coated to a thickness of 25 nm and thermally annealed in a vacuum oven at 150 °C for 14 h.

### Reactive-ion etching

Reactive-ion etching of the annealed block copolymer film was performed in two steps. First, the top PDMS wetting layer was removed using a 5 s CF_4_ plasma treatment with a power of 50 W and pressure of 15 mTorr. Next, the PS matrix was removed using a 22 s O_2_ plasma treatment with a power of 90 W and pressure of 6 mTorr. This step also oxidized the PDMS cylinders. For 16 kg/mol PS-*b*-PDMS, the CF_4_ plasma was applied for 3 s and O_2_ plasma was applied for 12 s.

### Metrology

Metrology was performed by examining the HSQ templates and the reactive-ion etched block copolymer films using a SEM. Top down SEM images were obtained using a Raith 150 SEM operated at 10 kV acceleration voltage and 6 mm working distance, and Zeiss Sigma SEM operated at 3 kV acceleration voltage and 4 mm working distance.

### Simulation details

Simulation details are provided in the additional file.

## Additional file

**Additional file 1.** Additional Figures S1–S7 and Simulation Details.

## Abbreviations

DSA: directed self-assembly
PS-*b*-PDMS: poly(styrene-*block*-dimethylsiloxane)
SD45: 45 kg/mol PS-*b*-PDMS
HSQ: hydrogen silsesquioxane
SEM: scanning electron microscope
SCFT: self-consistent field theory
PGMEA: propylene glycol monomethyl ether acetate
