# Supplementary material for: Directed self-assembly of a two-state block copolymer system
Source: Nano Converg. 2018 Sep 27;5:25. doi: 10.1186/s40580-018-0156-z (PMC6158142; doi:10.1186/s40580-018-0156-z)
Supplement: Supplementary file 1 — Additional file 1. Additional Figures S1–S7 and Simulation Details. [file 40580_2018_156_MOESM1_ESM.pdf]

Additional File

Directed Self-Assembly of a Two-State Block Copolymer System

Hyung Wan Do[a,b], Hong Kyoon Choi[c,d], Karim R. Gadelrab[c], Jae-Byum Chang[c,e], Alfredo Alexander-Katz[c], Caroline A. Ross[c], Karl K. Berggren[a]*

[a]Department of Electrical Engineering and Computer Science, Massachusetts Institute of Technology, Cambridge, MA 02139, USA

[b]Department of Chemical and Biomolecular Engineering, Korea Advanced Institute of Science and Technology, Daejeon, Korea

[c]Department of Materials Science and Engineering, Massachusetts Institute of Technology, Cambridge, MA 02139, USA

[d]Division of Advanced Materials Engineering, Kongju National University, Cheonan, Korea

[e]Department of Biomedical Engineering, Sungkyunkwan University, Seoul, Korea

*corresponding author, berggren@mit.edu

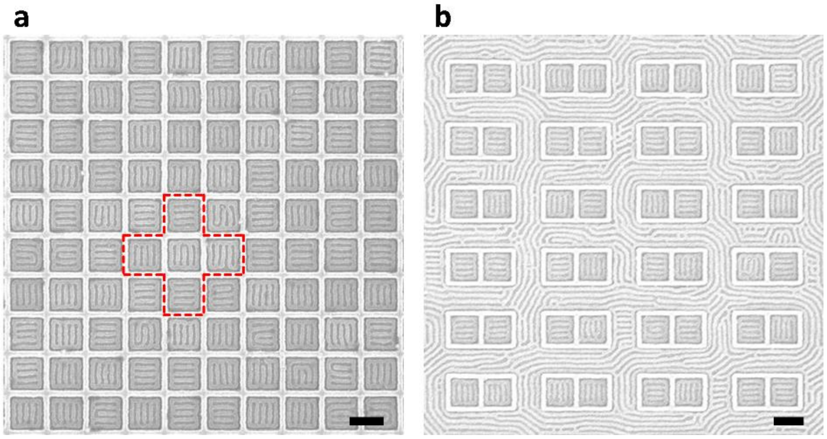

Figure S1. SEM images of ladder-shaped block copolymer patterns inside square confinement for measuring distribution and correlation. (a) The two binary states were equally probable in a large array of square confinements. (b) The two states were uncorrelated in pairs of adjacent square confinements. For both figures, width of the square confinement was $5.1L_0$, resulting in PDMS patterns with 3 parallel bars inside an outer ring. Scale bars, 200 nm.

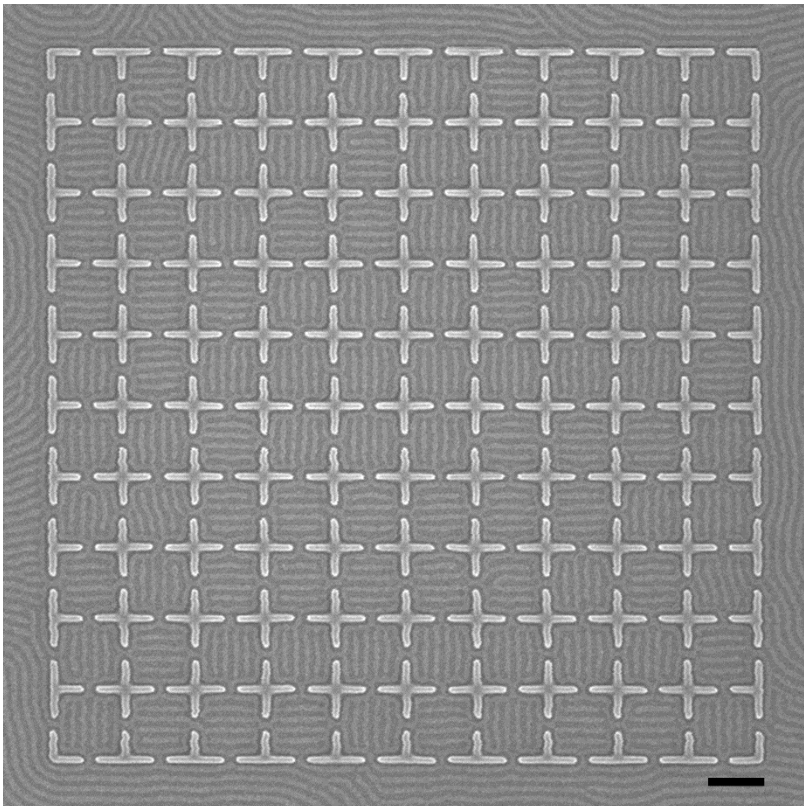

Figure S2. SEM image of a 10 by 10 array of square confinements with four openings. Due to the defect tolerance of the system, the binary state inside each confinement can be uniquely determined even in the presence of defects. Scale bar, 200 nm.

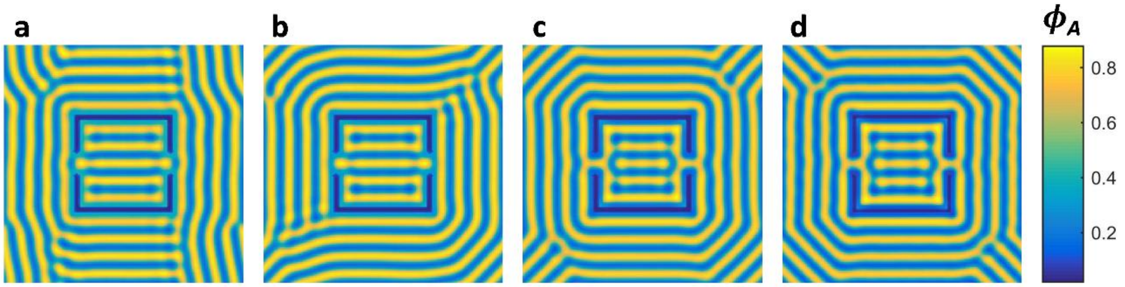

Figure S3. SCFT simulations showing the density distribution of block *A* for different wetting conditions w_-. The higher the magnitude of w_-, the stronger the attraction to *A* at the walls. (a) $w_- = 1.5$, (b) $w_- = 2.5$, (c) $w_- = 4.5$, and (d) $w_- = 5.5$. The wetting at the walls does not affect the final state of the self-assembled polymer, indicating the effectiveness of having two opposite openings in directing the self-assembly of block copolymer. The experimental results are reproduced for $2.5 < w_- < 5.5$.

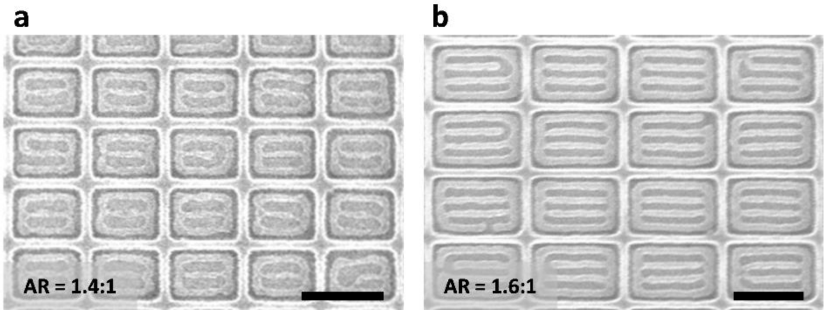

Figure S4. SEM images of aligned ladder-shaped block copolymer patterns inside rectangular confinement with a non-integer aspect ratio (AR). Vertical width of the rectangular confinement was (a) $2.9L_0$ and (b) $4.1L_0$, approximately commensurate with the equilibrium periodicity. Horizontal width of the confinement was (a) $4.1L_0$ and (b) $6.6L_0$. Aspect ratio was (a) 1.4:1 and (b) 1.6:1. Scale bars, 200 nm.

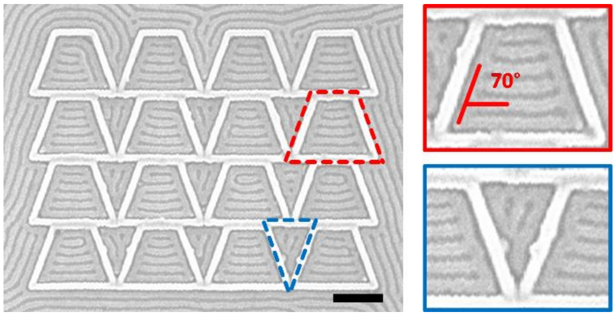

Figure S5. SEM images of aligned ladder-shaped block copolymer patterns inside trapezoidal (red) and isosceles triangular (blue) confinement. The ladder-shaped structures were typically aligned parallel to the longer side to minimize T-junction formations. Vertical width of the confinement was $5.8L_0$. Scale bar, 200 nm.

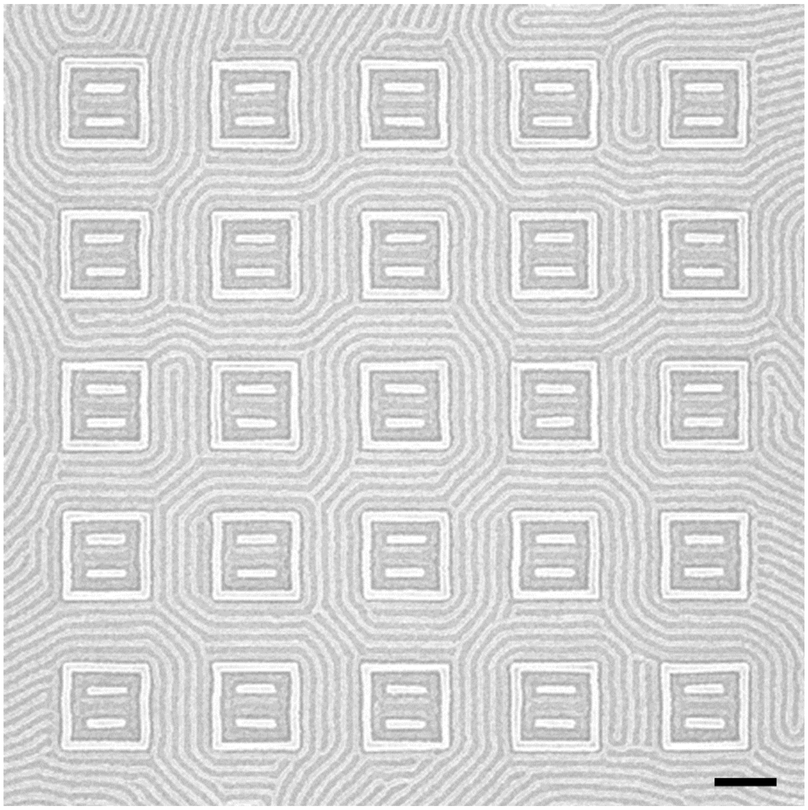

Figure S6. SEM image of aligned ladder-shaped block copolymer patterns inside square confinement with horizontal guiding patterns. Without the guiding patterns, ladder-shaped structures with four horizontally or vertically aligned PDMS bars were formed with equal probability. However, by placing functionalized HSQ walls where the PDMS bars should form, alignment orientation was controlled. Scale bar, 200 nm.

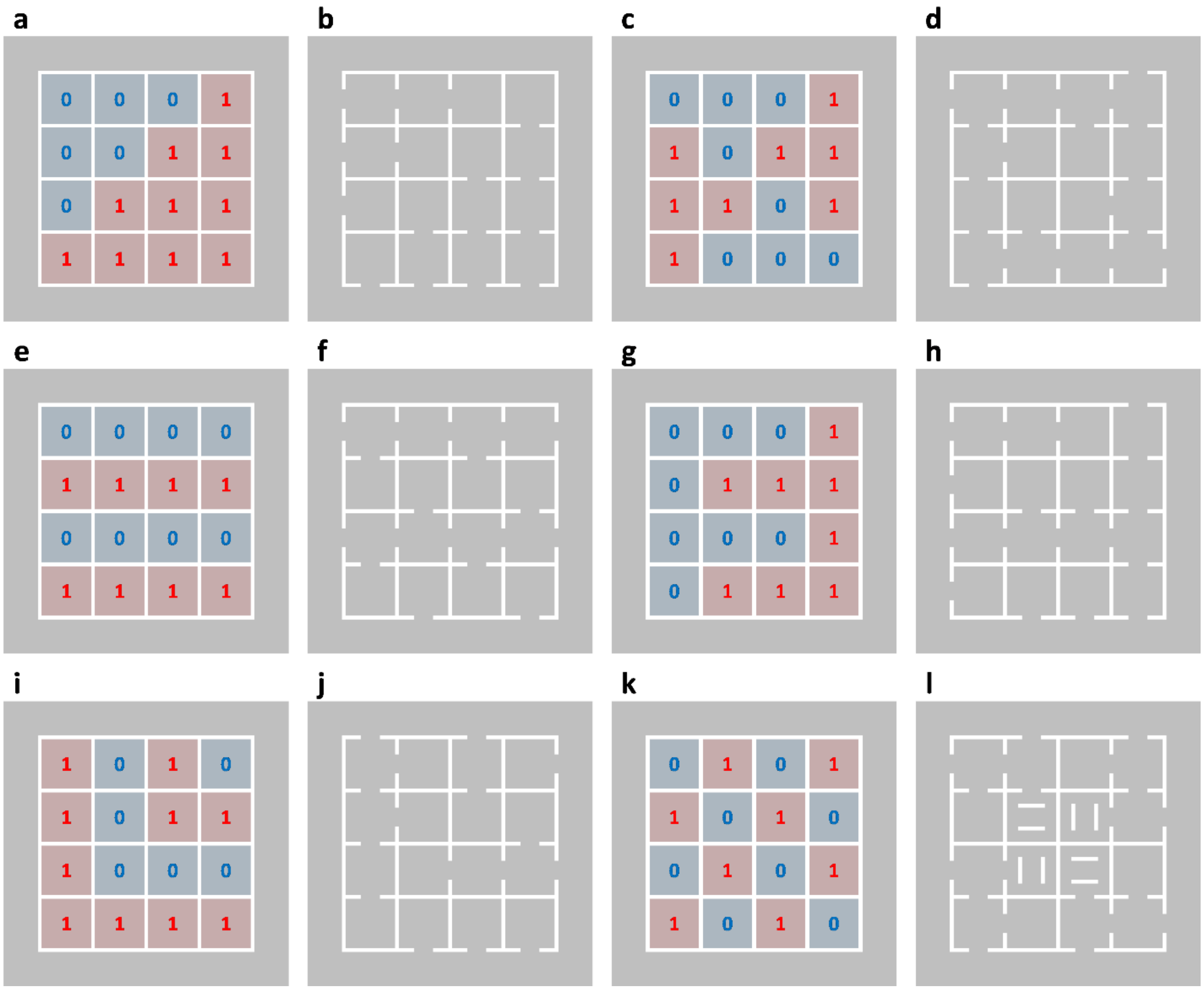

Figure S7. Examples of target patterns and corresponding template designs. (a,c,e,g,i,k) Diagram of desired 4 × 4 binary state arrays. (b,d,f,h,j,l) Template designs that will produce the target patterns. Figure S7b and Figure S7d show alternative template designs for the same target patterns from Figure 6. Figure S7k shows a target pattern unobtainable from creating openings in the walls. Figure S7l shows a template design consisting of both openings and guiding patterns.

Simulation details

We consider a monodispersed melt of n A-B diblock copolymer of volume V, with each diblock molecule composed of N segments. The A and B blocks consist of fN and $(1 - f)N$ chain segments, respectively. The interaction between the dissimilar blocks is controlled by a Flory-Huggins parameter χ. Within the mean-field approximation, the free energy of the system F is expressed in terms of field variables

$$\frac{F}{nVk_{\mathrm{B}}T} = \frac{1}{V}\int dr(\chi\phi_A(r)\phi_B(r) - w_A(r)\phi_A(r) - w_B(r)\phi_B(r) - p(r)[1 - \phi_A(r) - \phi_B(r)]) - \ln Q[w_A, w_B]$$

where $\phi_\alpha(r)$ is the volume fraction of species α at position r. $Q[w_A, w_B]$ is the partition function of a non-interacting polymer in external fields $w_\alpha(r)$. The polymer is assumed to be incompressible, so the constraint $\phi_A(r) + \phi_B(r) = 1$ is enforced through a pressure field $p(r)$. The free energy F is compared to the thermal energy $k_{\mathrm{B}}T$.

The single chain partition function can be evaluated as follows

$$Q = \frac{1}{V}\int drq(r, 1)$$

where $q(r, s)$ is a restricted chain partition function (propagator) that could be calculated by solving a modified diffusion equation

$$\frac{\partial q}{\partial s} = \nabla^2 q(r,s) - w_A(r)q(r,s), \qquad 0 \le s < f$$

$$\frac{\partial q}{\partial s} = \nabla^2 q(r,s) - w_B(r)q(r,s), \qquad f \le s < 1$$

subjected to the initial condition $q(r, 0) = 1$. Since the two ends of the polymer are distinct, a complementary partition function $q^*(r, s)$ is defined similarly and satisfies the same modified diffusion equation with an initial condition $q(r, 1) = 1$. Here, we utilize s as a chain contour variable

in units of N. All lengths are expressed in units of the unperturbed radius-of-gyration of a polymer, $R_g = (Nb^2/6)^{1/2}$, where b is the statistical segment length. The solution to the modified diffusion equation is conducted following the pseudo-spectral method.[1] An iterative relaxation of the fields towards their saddle-point values is implemented following the method by Sides et al.[2]

By evaluating $q(r, s)$ and its complementary, the segments' volume fractions can be determined as follows

$$\phi_A(r) = \frac{1}{Q}\int_0^f ds q(r,s) q^*(r,s)$$

$$\phi_B(r) = \frac{1}{Q}\int_f^1 ds q(r,s) q^*(r,s)$$

The numerical implementation of the SCFT for the entire template is performed on a 2D square grid of size $N_x = N_y = 240$ pixels (pixel size is $0.2R_g$) with periodic boundary conditions in both directions. The volume fraction f and degree of incompatibility χN are chosen such that striped domains (projection of in-plane cylinders in 2D) are generated. Hence, for the purpose of this work, we chose $f = 0.5$ and $\chi N = 12$.

The role of the DSA of the polymer domains is depicted through a masking method. A pressure potential $w_+ = (w_B + w_A)/2$ is imposed as a mask on the location of the walls to create excluded areas for the polymer. A magnitude of $w_+ = 10$ is applied on walls of thickness of six pixels. To incorporate the effect of surface preferentiality towards the majority block in experiments, an exchange potential $w_- = (w_B - w_A)/2 = 3.5$ is applied surrounding the walls with a thickness of four pixels to attract block B. The confining template is displaced from the boundaries of the computational domain by 70 pixels to minimize the effect of the mirror image (periodic boundary conditions) on the polymer domains near the walls.

References

1. G. Tzeremes, K. Ø. Rasmussen, T. Lookman, A. Saxena, *Phys. Rev. E* **65**, 4 (2002)
2. S. W. Sides, G. H. Fredrickson, *Polymer* **44**, 19 (2003)
